# Author’s Reply—Atropine: Hero or villain in cardioneuroablation?

**DOI:** 10.1016/j.hrcr.2022.05.007

**Published:** 2022-05-12

**Authors:** Clinton J. Thurber, Davis R. Sneider, William H. Sauer, Sunil Kapur

**Affiliations:** ∗ Brigham and Women’s Hospital, Boston, Massachusetts; † Abbott Laboratories, 100 Abbott Park Rd, Abbott Park, Illinois

We thank Drs Pachon for their interest in our recent report. Collaboration is critical in novel procedures.

The comment “atropine at CNA beginning was the villain” is unclear; there was no “rapid innervation.” Rather, we objectively demonstrated persistent denervation. Fundamentally, an interesting observation of our report is not the recurrence itself, but the nature of the recurrence. Heart rate (HR) and HR variability slowly normalizing over an extended time course does not suggest a simple procedural failure, but perhaps that more complex physiology is at play.

In this early stage, no cardioneuroablation (CNA) protocols have garnered sufficient evidence for widespread adoption. We recognize a school of thought advocating for earlier procedural atropine administration, as acknowledged in our report. However, during the procedure, the patient’s HR drifted back down to the upper 60 beats per minute range, near the baseline in the upper 50s. As such, both the subsequent 36% HR increase produced by radiofrequency delivery and the blunted HR response to postablation atropine remain reliable indicators of efficacy. Further, our lab has since generated unpublished data from a repeat atropine challenge in this same patient 10 months later, in which no HR increase could be provoked. It is not clear based on available data how earlier atropine administration would have changed the localization or ablation of ganglionated plexi.

“Abandonment” of high-frequency stimulation has not been prevalent, less so at the time of this procedure early in 2021, and labs that exclude high-frequency stimulation generally do so in favor of an electrogram-only approach, rather than replacing it with extracardiac vagal stimulation, as implied.^1^, ^2^, ^3^ We are interested in the potential of extracardiac vagal stimulation to optimize denervation assessment; however, its incorporation in mainstream CNA workflows has been, as Drs Pachon cite, quite limited.

In addition, the typographical error has been corrected, and the anatomic sites in question were indeed covered by the initial lesion set.
